# The Benefits of an Integral HAMMAM Experience Combining Hydrotherapy and Swedish Massage on Pain, Subjective Well-Being and Quality of Life in Women with Endometriosis-Related Chronic Pelvic Pain: A Randomized Controlled Trial

**DOI:** 10.3390/medicina60101677

**Published:** 2024-10-13

**Authors:** Ángel Rodríguez-Ruiz, Camila Arcos-Azubel, Manuel Ruiz-Pérez, Francisco Manuel Peinado, Antonio Mundo-López, Ana Lara-Ramos, María del Mar Salinas-Asensio, Francisco Artacho-Cordón

**Affiliations:** 1 Department of Radiology and Physical Medicine, University of Granada, E-18016 Granada, Spain; angel2002@correo.ugr.es (Á.R.-R.); camilarcos@correo.ugr.es (C.A.-A.); manuruiz@correo.ugr.es (M.R.-P.); 2 Biohealth Research Institute in Granada (ibs. GRANADA), E-18012 Granada, Spain; franciscopeinado@correo.ugr.es; 3 Clinic Psychology Center Alarcón (CPCA), E-18004 Granada, Spain; antonio@alarconpsicologos.com; 4 Gynecology and Obstetrics Unit, ‘Virgen de las Nieves’ University Hospital, E-18016 Granada, Spain; ana_lara_ramos@hotmail.com; 5 Department of Physiotherapy, University of Granada, E-18016 Granada, Spain; marsalinas@ugr.es; 6 CIBER Epidemiology and Public Health (CIBERESP), E-28029 Madrid, Spain

**Keywords:** endometriosis, hydrotherapy, Swedish massage, pain intensity, pain interference, pain threshold

## Abstract

*Background and Objectives*: To evaluate the effectiveness of an integral HAMMAM experience, a 4-week therapeutic program that combined hydrotherapy and Swedish massage, applied in a multisensorial immersive environment, on pain, well-being and quality of life (QoL) in women with endometriosis-related chronic pelvic pain that is unresponsive to conventional treatment. *Materials and Methods*: This randomized controlled trial included 44 women with endometriosis. They were randomly allocated to either the ‘HAMMAM’ group (*n* = 21) or to a control group (*n* = 23). The primary outcome, pain intensity, was evaluated using numeric rating scales (NRSs). The secondary outcomes were pain interference, pain-related catastrophic thoughts, pressure pain thresholds (PPTs), subjective well-being, functional capacity and QoL, which were evaluated using the brief pain inventory (BPI), the pain catastrophizing scale (PCS), algometry, the subjective well-being scale-20 (EBS-20), the Patient-Reported Outcomes Measurement Information System-29 (PROMIS-29) and the Endometriosis Health Profile-30 Questionnaire (EHP-30), respectively. The primary and secondary outcomes were measured at the baseline and after the intervention. The statistical (between-group analyses of covariance) and clinical effects were analyzed by the intention to treat. *Results*: The adherence rate was 100.0% and the mean (± standard deviation) satisfaction was 9.71 ± 0.46 out of 10. No remarkable health problems were reported during the trial. The ‘HAMMAM’ intervention improved dysmenorrhea and dyspareunia after the intervention with large and moderate effect sizes, respectively. Improvements in pain interference during sleep and PPTs in the pelvic region were also observed in women allocated to the ‘HAMMAM’ group. No effects were observed in catastrophizing thoughts, well-being nor QoL, except for the sleep subscale. *Conclusions*: A 4-week program of an integral ‘HAMMAM’ experience combining hydrotherapy and massage in a multisensorial immersive environment is a feasible and effective intervention to alleviate pain during menstruation and sexual intercourse as well as pain interference with sleep in women with endometriosis.

## 1. Introduction

Endometriosis is among the most prevalent gynecologic diseases with estimated prevalence rates ranging 5–15% worldwide [1]. Characterized by the presence of endometrial-like tissue outside the uterine cavity, this complex condition causes a vast range of symptoms in suffering women [2], including both physical and mental problems, which in turn, diminishes their quality of life (QoL) [3,4,5]. 

It is acknowledged that pain, located in the pelvic region, is the most common, persistent and debilitating symptom in women diagnosed with endometriosis, which usually increases during menstruation (dysmenorrhea) or daily activities such as sexual relationships (dyspareunia), defecation (dyschezia) and/or urination (dysuria). Moreover, the chronic nature of endometriosis-related pain leads to sensitization of the local region and to the development of central sensitization signs (CCSs) [4,6]. In addition, women with endometriosis experiences report catastrophizing thoughts related to pain as well as elevated rates of stress, anxiety, depression and sleep disorders [5]. Therefore, this endometriosis-related symptom burden is responsible for reductions in the performance of daily life activities and work, and thus, reductions in QoL and well-being [7,8]. 

During these last decades, the conventional management for endometriosis usually includes medical treatment (analgesics, oral contraceptives, etc.) and surgical interventions for selected cases. However, it has been estimated that 30–60% of patients report that their symptom burden amelioration after treatment is null or insufficient [9]. Thus, given the elevated failure rates of medical treatment, complementary therapeutic approaches that may help to reduce the symptom burden are being explored in recent years. For instance, pelvic floor physiotherapy (including manual therapy and exercise) [10] as well as global exercise-based therapy [11], massage [12] and manual therapy [13] interventions have shown to be effective to reduce pain and improve the QoL in those women with endometriosis that is unresponsive to conventional treatment. Moreover, some electrophysical agents such as transcutaneous electrical nerve stimulation (TENS) [14] or deep thermotherapy applied through radiofrequency diathermy [15] have also shown benefits for women with endometriosis. In this regard, heat is known to decrease pain in soft tissues by accelerating tissue metabolism, blood flow, tissue healing and connective tissue extensibility, among other effects [16,17,18]. Heat can be applied through multiple tools, although the use of heat applied through water might be of interest in women with endometriosis, as some studies have confirmed the effectiveness of this therapeutic tool in a variety of chronic pain-related conditions [19,20,21]. Moreover, the combination of different therapeutic strategies may enhance the benefits exerted by the individual therapeutics. 

Thus, the aim of this study was to explore the short-term effects on pain, well-being and QoL of a 4-week integral experience in an ancient HAMMAM Arab bath where hydrotherapy, combined with massage, is applied in a multisensorial immersive environment in women with endometriosis-related chronic pelvic pain (CPP) that is unresponsive to conventional treatment.

## 2. Materials and Methods

### 2.1. Study Design and Participants

A parallel group-randomized controlled trial (RCT) (ClinicalTrials.gov, NCT06506708) was conducted between 2023 and 2024, recruiting 44 women with endometriosis-related CPP from ‘Virgen de las Nieves’ public University Hospital in Granada (Spain). Inclusion criteria were as follows: women 20–50 years of age diagnosed with endometriosis (confirmed by surgery, magnetic resonance imaging, or ultrasound imaging), presence of pelvic pain during the past 6 months with a score of 4 or higher on a 0–10 NRS scale and a period of at least 3 months since the last surgery. Additional criteria included premenopausal status, the ability to walk without assistance, adequate literacy skills and being capable and willing to provide informed consent. Exclusion criteria were as follows: acute or terminal illness, a recent fracture in any upper or lower extremity (<3 months), disc herniation and any chronic disease or orthopedic issues that would interfere with her ability to participate in this intervention program, expressed unwillingness to complete the study requirements or involvement in other rehabilitation programs. 

This trial adheres to the CONSORT 2010 statements [22]. All participants provided written informed consent. This study was approved by the Clinical Research Ethics Committee of Granada, Government of Andalusia, Spain (code: 2031-N-22).

### 2.2. Randomization

Participants who met the eligibility criteria were assigned to either the ‘HAMMAM’ or control group through a simple computer-generated randomization sequence, handled by a researcher not involved in the clinical part of the study to ensure that the assessors remained blinded to the randomization. Once the baseline assessments were completed, the principal investigator opened the opaque, numbered envelopes to reveal and communicate the group assignments to the participants. As a result, although the practitioners and participants were aware of the group assignments, the other researchers, including assessors, statisticians, and data managers, were blinded to these allocations. Participants were also instructed to refrain from discussing any details of their treatment or group assignment with the assessors conducting the evaluation sessions.

### 2.3. Intervention

The intervention was accomplished at the Arab baths of Hammam Al Ándalus, Granada (Spain). Over a period of four weeks, equivalent to the length of a menstrual cycle, participants participated in an integral HAMMAM experience, which was divided into three sessions of 1.5 h each, separated by an interval of 14 days. Each session begun with a thermal circuit in which, following the institution’s guidelines, women were invited to explore the warm (33–34 °C), hot (38 °C), and cold-water (17–19 °C) baths, as well as the steam room and the hot stone table during a period of 60–75 min. The thermal circuit was complemented by a 15-min full-body Swedish massage using essential oils that included rubbing, kneading, stroking and tapping of the main muscles. During the last session, Swedish massage was preceded by a 15-min traditional Kessa massage. During Kessa massage, women lied on a hot stone table for 15 min, while the skin was covered with red grape soap suds and scrubbed with a cotton fiber glove (kessa). The scrubbing cleansed the skin of any dirt and residues, helping women to achieve a fully relaxed feeling. Swedish and Kessa massages were performed by trained massage therapists with some years of experience.

This entire process was also accompanied by a multisensorial immersive experience, as intervention was accomplished at an ancient HAMMAM Arab bath in Granada (Spain), a building all decorated in resplendent Arabian décor, which combined lighting and a palette of delicate colors, exotic aromas, tea tasting and relaxing music, bringing into play the senses of sight, smell, taste and hearing. 

As with the intervention group, the control group continued with the medical treatment prescribed by their gynecologist. Additionally, during the evaluation, women were given advice on the importance of following a healthy lifestyle to improve their QoL.

### 2.4. Outcome Measures

Data were collected at baseline and post-intervention. 

#### 2.4.1. Primary Endpoint

*Pain intensity.* It was considered the primary outcome of this study, which was assessed through a Numeric Rating Scale (NRS). It is a 11-Likert scale used for subjective pain estimation. It ranges from 0 (“no pain”) to 10 (“worst imaginable pain”). Participants were asked to indicate their current levels of CPP, dysmenorrhea, dyspareunia, dyschezia and dysuria by selecting the whole number that best reflects the intensity of the pain that they feel during basal and final evaluation sessions. The NRS has been widely used and has previously shown to be a reliable and valid instrument for assessing pain with an intraclass correlation coefficient (ICC) 0.95 [23], and it has been identified as the most appropriate tool for self-reported pain intensity assessment in endometriosis patients, given that although both visual analogue scales (VASs) and NRSs are valid, reliable and precise scales, NRS is easier to fulfill and administer compared with VAS [24].

#### 2.4.2. Secondary Endpoints

*Pain interference.* It was addressed through the corresponding subscale of the Brief Pain Inventory (BPI). It measures how much pain has interfered with seven daily activities, including general activity, walking, work, mood, enjoyment of life, relations with others and sleep. Scores are obtained on a scale from 0 to 10 where 0 is ‘no interference’ and 10 means ‘complete interference’. BPI pain interference is typically scored as the mean of the seven interference items, although pain interference in each evaluated activity can be interpreted individually. This approach has good to excellent validity and reliability for assessing interference in chronic pain subjects [25,26], with good overall internal consistency (Cronbach’s alpha: 0.87) and excellent test–retest reliability (ICC 0.96), as well as inter-rater reliability (ICC: 0.77) [27].

*Catastrophic thoughts related to pain.* They were assessed through the Spanish version of the PCS, a 13-item, validated, self-report instrument with adequate reliability (Cronbach’s alpha 0.79) [28]. This measure has a 5-point Likert-style response scale, and the scoring range is 0–52, with higher scores indicating higher levels of catastrophic thoughts. The PCS has shown good reliability (Cronbach’s alpha 0.95) [29].

*Pressure pain thresholds (PPTs).* To determine the measurements, an electronic algometer was utilized (JTECH Medical algometer Commander Echo, JTECH, Riverton, UT, USA). The pressure was applied at an approximate rate of 0.3 kg/s using a 1 cm² probe. For the main analysis, the mean of three trials, with a 30-s resting period between trials, was calculated. Pressure algometry demonstrated an intraclass correlation coefficient (ICC) of 0.91 and a minimum clinically important difference of approximately 174 kPa [30]. As previously reported, a total of 11 points were tested across four different regions: abdominal, pelvic, lower back and the second metacarpals as distal points [4]. In the abdominal wall, four points were marked bilaterally. The supraumbilical points were assessed 3 cm above the umbilical point within the hemiclavicular line (the lateral border of each rectus muscle). The infraumbilical point was assessed 3 cm below the umbilical point within the same line. The pelvic region was evaluated at three additional points: the pubic symphysis and both inguinal ligaments at their midpoint. The lower back region was evaluated bilaterally, using the spinous process of the fifth lumbar vertebra as a reference, with the algometer placed in the paraspinal area, in the middle of the erector spinae muscle (approximately 3 cm to the right or left of the marked spine). Finally, the second metacarpals on both sides were assessed as a distant point from the affected area. Additionally, PPTs from each region (abdominal, pelvic and low back regions, and second metacarpals as distal points) were calculated as the mean of the PPTs from this region.

*Subjective well-being.* It was assessed through the Subjective Well-being Scale (EBS-20), a self-administered tool used to assess satisfaction and positive affect toward life. It is made up of 20 6-point Likert-type items. The items are divided into 2 subscales [satisfaction with life (SV) and positive affect (PA)], scoring each item from 0 to 6. The score for each subscale is obtained as the mean of the item scores from each subscale, while the total score is calculated as the mean of all EBS-20 items. Higher scores indicate greater satisfaction or positive affect in the lives of the patients. The Spanish version was validated and showed evidence of content, structure, criterion and reliability validity, with a Cronbach’s alpha >0.96 for both subscales [31].

*Functional capacity and QoL.* They were assessed with the Patient-Reported Outcomes Measurement Information System-29 (PROMIS-29) and the Endometriosis Health Profile-30 Questionnaire (EHP-30). PROMIS-29 is a self-administered scale that aims to evaluate the functionality and QoL of patients with a wide variety of clinical situations. It consists of 29 5-point Likert-type items that are divided into eight subscales, which are in turn organized into 3 blocks: physical functioning capacity, fatigue, sleep disturbance, effects of pain and pain intensity (physical health), anxiety and depression (mental health) and ability to participate in social roles and activities (social health). The scores obtained in each subscale range from 4 (minimum) to 20 (maximum) except for pain intensity, in which its values range from 0 to 10 points. Regarding the interpretation of the results, higher scores indicate worse functionality, except for the subscales of physical functioning capacity and ability to participate in social roles and activities, where higher scores indicate better functionality [32]. It has been shown to be reliable and valid in patients with pathologies of physical origin, with a Cronbach’s alpha coefficient of 0.98 [32]. The EHP-30 scale is a specific tool to assess QoL in patients with endometriosis. This self-administered questionnaire consists of 30 Likert-type items of 5 points (0 to 4) organized into 5 subscales: pain (11 items), control and helplessness (6 items), emotional well-being (6 items), social support (4 items) and self-image (3 items). The score on each subscale is obtained as a percentage, fluctuating between 0 (best health status) and 100 (worst health status). The total score is obtained through the percentage obtained considering the 30 items [33]. The Spanish version has shown great validity and reliability (Cronbach’s alpha 0.79–0.97) [34].

### 2.5. Statistical Analysis

In the descriptive analysis, continuous variables were expressed as means ± standard deviations (SDs) and categorical variables as percentages. The Shapiro–Wilk test was used to test the normal distribution of the data (*p* > 0.050). Bivariate comparisons to detect between-group differences at baseline used the chi-square (or Fisher’s exact) test and Student’s (or Mann–Whitney) test as appropriate. 

Formal analysis was accomplished on an intention-to-treat (ITT) basis. To examine the influence of treatment on outcome scores, a 2-way repeated-measures analysis of covariance (ANCOVA) with intervention (HAMMAM/control) as the between-group variable and time (baseline/post-intervention) as the within-group variable were used. No adjustment was required given that none of the variables showed significant differences between groups at baseline. Effects on study variables of the intervention and the persistence of these effects were calculated, and the effect size (Cohen d) was estimated, and classified as negligible (<0.2), small (0.2–0.5), moderate (0.5–0.8) or large (>0.8). No missing data was observed, and therefore, multiple imputations were not needed. The adherence rate was the ratio of the number of sessions performed to the number of sessions prescribed. 

The significance level was set at *p* < 0.050, although results with *p*-values between 0.100 and 0.050 were also cautiously discussed. Analyses were performed using SPSS v28.0 statistical software (IBM, Chicago, IL, USA). 

To detect a minimum of 1.25-point difference (SD 1.5) in the NRS scale for CPP [35] between the groups, with an alpha value of 0.05, and assuming a 95% statistical power, 22 participants per group were needed (i.e., 44 participants in total), assuming a 10% dropout rate. Sample size calculations were performed with G*Power v3.1.9.7 software (Düsseldorf, Germany).

## 3. Results

**Figure 1:**
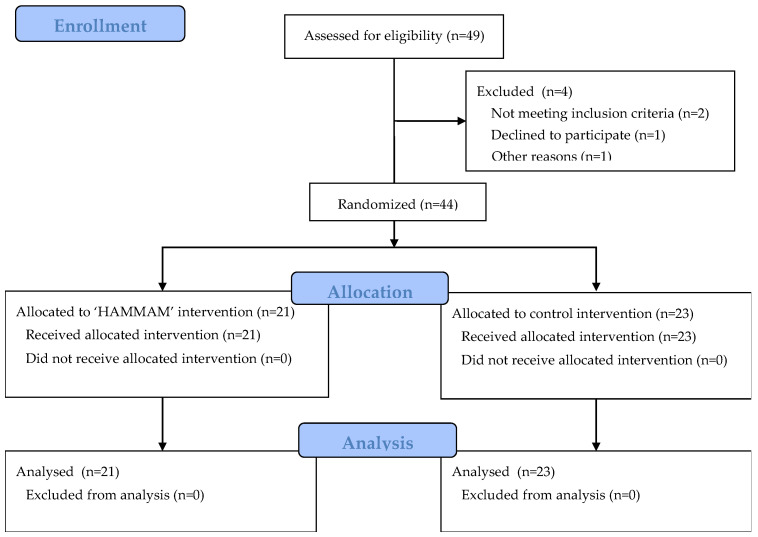
The flow of participants in the trial.

Forty-four women met the inclusion and exclusion criteria and were randomly assigned to the ‘HAMMAM’ (*n* = 21) or control groups (*n* = 23) (Figure 1). The sociodemographic and clinical characteristics of the study population are summarized in Table 1, while the study outcome scores at baseline are depicted in Table 2. As shown, there were no between-group differences in the sociodemographic or clinical characteristics, nor in the study variables at baseline. All participants attended all of the three prescribed sessions, representing an adherence rate of 100.0%. None of the participants reported any remarkable health problems during the trial. The mean satisfaction with the ‘HAMMAM’ program, evaluated by an NRS at the end of the intervention, was 9.71 ± 0.46 out of 10.

### 3.1. Primary Outcome: Pain Intensity

Time × group interactions were found for dysmenorrhea (F = 4.852, *p* = 0.035) and dyspareunia (F = 5.853, *p* = 0.020), while no interactions were observed for pelvic pain, dyschezia or dysuria (Table 3). The post-hoc ANCOVAs showed significant post-intervention improvements in dysmenorrhea and dyspareunia in the ‘HAMMAM’ group (*p* < 0.050 for both). A large effect size was found for the between-group differences post-intervention in dysmenorrhea (d = 0.80; 95%CI 0.35 to 1.25) and a moderate effect size for dyspareunia (d = 0.62; 95%CI 0.03 to 1.21).

### 3.2. Secondary Outcomes

#### 3.2.1. Other Pain-Related Outcomes

With respect to the results related to the BPI, a significant time × group interaction was not observed for overall pain interference in daily activities nor in catastrophic thoughts related to pain (Table 4). However, when pain interference in each activity was analyzed, a significant time × group interaction was identified for the interference of pain with sleep (F = 6.103, *p* = 0.018) (Table S1). A post-hoc ANCOVA showed a significant post-intervention reduction in pain interference with sleep in the ‘HAMMAM’ group (−1.84 [−2.29, −0.33]). A moderate effect size was found for the between-group differences post-intervention in pain interference with sleep (d = 0.74; 95%CI 0.00 to 1.49).

Regarding algometry, the results indicated a significant time × group interaction for the mean PPTs of the pelvic region (F = 5.098, *p* = 0.029), while no interactions were observed for those from the abdominal and lower back regions (Table 4). The post-hoc ANCOVAS showed significant post-intervention improvements in PPTs from the pelvic region in the ‘HAMMAM’ group (*p* < 0.050). Furthermore, considering the individualized PPTs from each analyzed point, significant time × group interactions were observed for the left inguinal ligament (F = 8.559, *p* = 0.006), while the interaction was close to statistical significance for both the pubic symphysis (F = 2.808, *p* = 0.083) and the right inguinal ligament (F = 2.963, *p* = 0.093) (Table S2). Similarly, significant post-intervention improvements were observed in the ‘HAMMAM’ group in PPTs from the left inguinal ligament, while improvements in the ‘HAMMAM’ group did not reach the statistical significance in PPTs from the pubic symphysis (0.40 [−0.05–0.85]; *p* = 0.083) nor the right inguinal ligament (0.32 [−0.06–0.70]; *p* = 0.093). In relation to the PPTs at regions distal to the affected area, no significant time × group interaction was observed in the metacarpal region (Table 4), although the PPT in the left metacarpal point showed a close to statistical significance time × group interaction (F = 3.030, *p* = 0.089). The post-hoc ANCOVAs revealed a close-to-significant improvement in the ‘HAMMAM’ group in the PPT at the left metacarpal point (0.48 [−0.08, 1.04], *p* = 0.089) (Table S2). A moderate effect size was found for the between-group differences post-intervention in PPTs for the overall pelvic region (d = 0.70; 95%CI 0.51 to 0.89) and a large effect size for the PPT in the left inguinal ligament (d = 0.91; 95%CI 0.72 to 1.10).

#### 3.2.2. Subjective Well-Being, Functional Capacity and QoL

In relation to the data obtained on the EBS-20 scale, no significant time × group interactions were observed, neither for the global scale, nor for the two subscales (Table 5).

Regarding the PROMIS-29 scale, the results indicated a significant time × group interaction for pain interference (F = 6.014, *p* = 0.018). Similarly, a close-to-statistical-significance time × group interaction was observed in the social health subscale (F = 3.146, *p* = 0.083). The post-hoc ANCOVAs showed significant post-intervention improvements in the pain interference subscale in the ‘HAMMAM’ group (−2.25 [−4.11, −0.40]), while the improvement in the ‘HAMMAM’ group for the social health subscale was close to statistical significance (1.09 [−0.28, 2.46], *p* = 0.083) (Table 5). A moderate effect size was found for the between-group differences post-intervention in pain interference (d = 0.76; 95%CI −0.12 to 1.64).

Finally, the results on the EHP-30 scale did not reveal significant time × group interactions, neither for the global scale, nor for the different subscales (Table 5).

## 4. Discussion

To the best of our knowledge, this study constitutes the first evidence of the benefits of a multisensorial immersive experience combining hydrotherapy and massage on endometriosis-related pain in women who are unresponsive to conventional treatment.

During recent years, a variety of studies have suggested the effectiveness of physical rehabilitative approaches to reduce endometriosis-related pain, including pelvic floor physiotherapy, therapeutic exercise or electrophysical agents. However, as clearly evidenced for other chronic pain conditions [36,37,38], the benefits of hydrotherapy, especially applied in combination with massage, have not been addressed in women with endometriosis yet. Moreover, hydrotherapy has been pointed out to be a cost-effective rehabilitation option compared to land-based therapy in patients with musculoskeletal disorders such as low back pain, osteoarthritis or rheumatoid arthritis, among others [39]. In this RCT, we clearly reported that this HAMMAM experience was enough to ameliorate pain intensity during menstruation and sexual intercourse, as well as to reduce pain interference during sleep, which is a daily life activity that is impaired in women with endometriosis [40,41]. To date, only a few studies have explored the isolated effectiveness of hydrotherapy or massage in patients with CPP related (or not) to endometriosis. For instance, despite not using tap water, our results are in line with those reported by Min et al. [35]. They observed that an intensive balneotherapy program (consisting of 10 sessions during 5 days of seawater baths at 38 °C for 20 min/session plus mud-pack applications at 40 °C for 10 min/session) was effective to reduce pain intensity and inflammatory markers in women with CPP. In this regard, the warmth of water can inhibit nociception by engaging thermal receptors and mechanoreceptors, thereby affecting spinal segmental mechanisms. Moreover, the heat can improve blood circulation and promote relaxation of pelvic floor muscles. The hydrostatic pressure may also contribute to the alleviation of pain by reducing the activity of the sympathetic nervous system [42,43,44]. Massage is another therapeutic tool that has been explored in women with chronic pelvic/low-back pain related (or not) to endometriosis [10,45,46,47,48,49,50,51]. However, the modality of the massage applied varies among these studies (Thiele massage, transverse friction massage or Swedish massage, among others), making comparisons with our results difficult. Nevertheless, in line with our findings, they unanimously reported benefits in terms of pain alleviation after massage. Although we did not explore the isolated effect of hydrotherapy nor Swedish massage on the studied outcomes, the combination of these two therapeutic tools was effective to reduce subjective pain intensity, in line with these previous studies. Moreover, we have identified, for the first time, that hydrotherapy, combined with massage, is also effective to improve pain sensitization in women with endometriosis-related CPP, measured through PPTs in the pelvic area, which is the most painful region in these women. In contrast with these previous studies exploring intensive programs (daily sessions), we observed significant positive effects on pain-related outcomes, even when the treatment was scheduled in a low-intensive program (three sessions over a month, approximately). However, in contrast with our study showing no effect on well-being nor QoL, some of these abovementioned studies also found positive effects on QoL. Nevertheless, despite the HAMMAM experience not showing a beneficial effect on overall QoL, an improvement in the sleep-related subscale was observed, suggesting that some aspects related to QoL were influenced by this combined therapeutic approach. Differences in the therapeutic tools applied, the frequency of the sessions, the evaluation tools used and the study population (it was not limited to women diagnosed with endometriosis but included those with CPP due to different reasons) may explain, at least in part, the discrepancies observed.

Moreover, HAMMAM experience also included aromatherapy (either within the essential oils applied for Swedish massage and or in environmental exotic aromas) and music therapy, which might have contributed to the benefits observed in this study. These findings are in line with those reported by Bakhtshirin et al. [52] showing that aromatherapy massage was beneficial for primary dysmenorrhea alleviation in young women or with those published by Merlot et al. [53] reporting pelvic pain reductions in women with endometriosis with an immersive digital therapeutic tool that included relaxing music (alpha/theta binaural beats, nature-based sounds). Similarly, music therapy has also been shown to be effective at ameliorating pain intensity in those undergoing oocyte retrieval treatments [54], and in both primary dysmenorrhea [55] and menstrual discomfort in young women [56].

It is important to note some limitations of our RCT. The primary weakness of this trial is the absence of study groups treated with isolated therapeutic tools (hydrotherapy or Swedish massage), which prevented us from elucidating the beneficial effect of each therapeutic approach used. Moreover, intra- and inter-variability in the professional that performed the massages could not be prevented, which might also influence the results observed. Additionally, this RCT had a limited sample size. Although we completed the estimated sample size to detect differences in the primary outcome (pain intensity), the size may have hindered the identification of subtle differences between groups in secondary outcomes such as well-being or QoL. Moreover, the simple randomization methodology followed for this small sample size led to an unequal distribution of individuals across groups. Nevertheless, a slight imbalance in group sizes due to simple randomization is unlikely to introduce bias, especially when baseline characteristics remain balanced and appropriate statistical adjustments are applied. Additionally, the eligibility criteria may limit the generalizability of our results to women with endometriosis. Furthermore, the variety of pharmacological treatments prescribed to women with endometriosis prevented us from homogenizing both groups based on this aspect. Thus, although there were no differences in the use of oral contraceptives between the groups and all women reported full compliance with the prescribed treatment, potential differences in the current treatment of the patients assigned to each group could also influence the results found.

## 5. Conclusions

Taken together, this study constitutes the first RCT that addresses the benefits of an integral HAMMAM experience combining hydrotherapy and Swedish massage in a multisensorial immersive environment on pain intensity and interference with daily life activities in women with endometriosis that is unresponsive to conventional therapy. Additionally, this study provided evidence for a safe therapeutic approach that can be added to the current treatments prescribed to symptomatic endometriosis patients. Nevertheless, further studies using additional study arms (testing isolated treatment options) and larger sample sizes should be carried out in the close future.

## Figures and Tables

**Table 1:** Demographic and clinical characteristics of participants.

	HAMMAM Group (*n* = 21)	Control Group (*n* = 23)	*p*-Value ^1^
	Mean ± SD	Mean ± SD	
**Age (yr)**	35.33 ± 6.65	35.26 ± 5.77	0.971
**Height (m)**	1.64 ± 0.05	1.64 ± 0.06	0.777
**Weight (kg)**	70.93 ± 10.40	71.72 ± 16.11	0.733
**Body mass index (kg/m^2^)**	26.30 ± 3.93	26.83 ± 6.41	0.832
**Marital status**			0.541
*Single*	12 (57.1%)	11 (47.8%)	
*Married/Divorced*	9 (42.9%)	12 (52.2%)	
**Employment status**			0.176
*Employed*	14 (66.7%)	20 (87.0%)	
*Unemployed*	2 (9.5%)	0 (0.0%)	
*Medical leave*	5 (23.8%)	3 (13.0%)	
**Educational level**			0.592
*Less than university*	8 (38.1%)	7 (30.4%)	
*University*	13 (61.9%)	16 (69.6%)	
**Smoking habits**			0.870
*No/Former smoker*	18 (76.2%)	16 (78.3%)	
*Yes*	5 (23.8%)	5 (21.7%)	
**Endometriosis diagnosis**			0.741
*Laparoscopy*	12 (57.1%)	12 (52.2%)	
*Imaging (MRI, US)*	9 (42.9%)	11 (47.8%)	
**Time since diagnosis (yr)**	5.12 ± 3.67	4.63 ± 4.30	0.397
**Number of endometriosis-related surgeries**			0.820
0	9 (42.9%)	12 (52.2%)	
1	9 (42.9%)	8 (34.8%)	
≥2	3 (14.3%)	3 (13.0%)	
**Oral contraceptives**			0.598
*No*	5 (23.8%)	4 (17.4%)	
*Yes*	16 (76.2%)	19 (82.6%)	
**Parity**			0.598
*Nulliparous*	16 (76.2%)	19 (82.6%)	
*Primiparous/multiparous*	5 (23.8%)	4 (17.4%)	

MRI: magnetic resonance imaging; SD: standard deviation; US: ultrasound imaging. ^1^ *p*-values for intergroup comparisons using the Mann–Whitney U test or Chi-square/Fisher exact test, as appropriate.

**Table 2:** Baseline scores for primary and secondary outcomes.

	HAMMAM Group (*n* = 21)	Control Group (*n* = 23)	*p*-Value
	Mean ± SD	Mean ± SD	
**Primary outcome**			
**Pain intensity—NRS**			
*Current pelvic pain*	5.41 ± 2.15	5.06 ± 1.71	0.469
*Dysmenorrhea*	7.24 ± 2.93	7.65 ± 1.17	0.660
*Dyspareunia*	6.71 ± 1.79	6.18±2.40	0.637
*Dyschezia*	5.65 ± 2.32	5.29 ± 2.44	0.499
*Dysuria*	3.82 ± 2.90	3.82 ± 2.53	0.981
**Secondary outcomes**			
**Pressure pain threshold—Algometry**			
*Supraumbilical, right side*	1.54 ± 0.69	1.55 ± 0.91	0.405
*Infraumbilical, right side*	1.27 ± 0.70	1.17 ± 0.75	0.440
*Supraumbilical, left side*	1.45 ± 0.73	1.50 ± 0.87	0.356
*Infraumbilical, left side*	1.31 ± 0.72	1.03 ± 0.64	0.126
*Pubis symphysis*	1.22 ± 0.62	1.10 ± 0.82	0.301
*Inguinal ligament, right side*	1.26 ± 0.83	1.06 ± 0.87	0.466
*Inguinal ligament, left side*	0.97 ± 0.52	1.00 ± 0.75	0.565
*Lumbar, right side*	2.01 ± 1.26	2.23 ± 1.47	0.630
*Lumbar, left side*	1.85 ± 1.28	2.30 ± 1.64	0.533
*Second metacarpal, right side*	2.17 ± 1.13	2.16 ± 1.12	0.396
*Second metacarpal, left side*	2.09 ± 1.29	2.21 ± 1.23	0.622
*Umbilical region*	1.39 ± 0.65	1.32 ± 0.72	0.304
*Pelvic region*	1.15 ± 0.67	1.05 ± 0.79	0.455
*Lumbar region*	1.93 ± 1.23	2.27 ± 1.54	0.565
*Metacarpals*	2.13 ± 1.17	2.19 ± 1.16	0.647
**Pain interference—BPI**			
*General activity*	4.76 ± 1.99	5.82 ± 2.68	0.344
*Mood*	5.59 ± 2.27	6.53 ± 1.97	0.305
*Walking ability*	4.00 ± 2.62	4.41 ± 2.98	0.501
*Normal work*	4.35 ± 2.57	4.82 ± 2.43	0.497
*Relationships with other people*	4.65 ± 2.69	4.35 ± 2.40	0.215
*Sleep*	5.41 ± 2.94	4.24 ± 2.41	0.105
*Enjoyment of life*	4.88 ± 2.85	5.71 ± 2.02	0.216
*Total*	5.15 ± 2.17	5.16 ± 2.13	0.497
**Pain catastrophizing thoughts—PCS**			
*Total*	27.62 ± 10.41	27.91 ± 11.33	0.888
**Subjective well-being—EBS-20**			
*Satisfaction with life*	3.30 ± 1.16	3.25 ± 1.00	0.436
*Positive affect*	3.79 ± 0.71	3.70 ± 0.78	0.345
*Total*	3.55 ± 0.85	3.48 ± 0.82	0.389
**Functioning—PROMIS-29**			
*Physical function*	16.00 ± 4.29	16.09 ± 3.36	0.905
*Pain intensity*	5.86 ± 2.15	5.96 ± 1.66	0.981
*Pain interference*	11.90 ± 3.55	11.96 ± 3.75	0.915
*Fatigue*	14.71 ± 3.54	15.00 ± 4.05	0.645
*Sleep disturbance*	13.57 ± 3.60	12.22 ± 3.25	0.098
*Anxiety*	11.95 ± 2.85	12.65 ± 3.42	0.234
*Depression*	10.81 ± 3.89	11.78 ± 3.42	0.191
*Ability to participate in social roles and activities*	11.24 ± 3.58	11.65 ± 3.50	0.350
**Quality of life—EHP-30**			
*Pain*	52.81 ± 19.75	57.61 ± 15.35	0.186
*Control and powerlessness*	59.72 ± 22.10	69.57 ± 18.18	0.056
*Emotional well-being*	55.75 ± 15.95	60.69 ± 15.93	0.155
*Social support*	59.23 ± 19.53	65.85 ± 18.89	0.130
*Self-image*	59.13 ± 19.53	67.75 ± 22.23	0.090
*Total*	56.27 ± 14.83	62.73 ± 12.60	0.063

BPI: Brief Pain Inventory; EBS-20: Subjective Well-Being-20; EHP-30: Endometriosis Health Profile-30; NRS: Numeric Rating Scale; PCS: Pain Catastrophizing Scale; PROMIS-29: Patient-Reported Outcomes Measurement Information System-29.

**Table 3:** Within- and between-group effects for pain intensity scores at baseline and post-intervention.

	HAMMAM Group (*n* = 21)	Control Group (*n* = 23)	Between-Group Effects
	Mean [CI(95%)]	Mean [CI(95%)]	
**Pain intensity—NRSW**			
**Current pelvic pain**			
*Baseline*	5.60 [4.38, 6.82]	5.06 [4.18, 5.94]	
*Post-intervention*	4.33 [2.74, 5.92]	4.88 [3.75, 6.02]	
*Within-group score change*	−1.27 [−2.77, 0.23]	−0.18 [−1.21, 0.86]	−1.20 [−2.70, 0.30]
**Dysmenorrhea**			
*Baseline*	7.13 [5.42, 8.84]	7.65 [7.05, 8.25]	
*Post-intervention*	5.87 [4.22, 7.51]	7.59 [6.88, 8.29]	
*Within-group score change*	−1.27 [−2.32, −0.21]	−0.29 [−0.65, 0.53]	−1.21 [−2.33, −0.09] *^b^
**Dyspareunia**			
*Baseline*	6.80 [5.77, 7.83]	6.18 [4.94, 7.41]	
*Post-intervention*	5.27 [3.71, 6.83]	5.88 [4.61, 7.15]	
*Within-group score change*	−1.53 [−2.97, −0.10]	−0.29 [−0.99, 0.40]	−1.24 [−2.71, −0.23] *^a^
**Dyschezia**			
*Baseline*	5.67 [4.33, 7.00]	5.29 [4.04, 6.55]	
*Post-intervention*	4.33 [2.61, 6.06]	5.00 [3.84, 6.16]	
*Within-group score change*	−1.33 [−2.85, 0.19]	−0.29 [−1.99, 1.40]	−1.27 [−2.88, 0.34]
**Dysuria**			
*Baseline*	4.27 [2.72, 5.81]	3.82 [2.52, 5.12]	
*Post-intervention*	3.20 [1.52, 4.88]	3.82 [2.48, 5.16]	
*Within-group score change*	−1.07 [−2.51, 0.38]	0.00 [−1.45, 1.45]	−0.81 [−2.30, 0.67]

Data are shown as mean [95% confidence interval for the mean] at baseline and post-intervention, and mean differences [95% confidence interval for the difference] for within- and between-group effects. Abbreviations: CI: confidence interval; NRS: numeric rating scale; Significant between-group effect * *p* < 0.05; ^a^ Moderate effect size: Cohen d 0.6–0.8; ^b^ Large effect size: Cohen d > 0.8.

**Table 4:** Within- and between-group effects for pain interference, pain catastrophizing thoughts and pressure pain thresholds at baseline and post-intervention.

	HAMMAM Group (*n* = 21)	Control Group (*n* = 23)	Between-Group Effects
	Mean [CI(95%)]	Mean [CI(95%)]	
**Pain interference—BPI**			
*Baseline*	5.08 [3.90, 6.25]	5.13 [4.16, 6.09]	
*Post-intervention*	3.94 [2.37, 5.51]	4.80 [3.46, 6.13]	
*Within-group score change*	−1.13 [−2.08, −0.18]	−0.33 [−1.58, 0.93]	−0.44 [−1.68, 0.80]
**Pain catastrophizing thoughts—PCS**			
*Baseline*	27.62 [22.88, 32.56]	28.17 [22.84, 33.51]	
*Post-intervention*	24.52 [18.92, 30.12]	25.61 [20.58, 30.64]	
*Within-group score change*	−3.10 [−7.81, 1.62]	−2.57 [−6.51, 1.38]	−0.56 [−7.54, 6.43]
**Pressure pain thresholds—Algometry**			
**Umbilical region**			
*Baseline*	1.41 [1.03, 1.79]	1.32 [0.95, 1.69]	
*Post-intervention*	1.51 [1.01, 2.01]	1.15 [0.78, 1.53]	
*Within-group score change*	0.10 [−0.31, 0.51]	−0.16 [−0.56, 0.23]	0.27 [−0.16, 0.71]
**Pelvic region**			
*Baseline*	1.10 [0.71, 1.49]	1.05 [0.65, 1.46]	
*Post-intervention*	1.49 [1.06, 1.92]	1.00 [0.61, 1.38]	
*Within-group score change*	0.39 [0.01, 0.77]	−0.06 [−0.38, 0.27]	0.42 [0.05, 0.80] *^a^
**Lumbar region**			
*Baseline*	1.92 [1.20, 2.64]	2.27 [1.48, 3.06]	
*Post-intervention*	2.40 [1.66, 3.14]	2.56 [1.46, 3.65]	
*Within-group score change*	0.48 [−0.14, 1.10]	0.29 [−0.39, 0.97]	0.30 [−0.47, 1.06]
**Metacarpals**			
*Baseline*	2.16 [1.47, 2.85]	2.19 [1.59, 2.78]	
*Post-intervention*	2.20 [1.45, 2.95]	2.16 [1.34, 2.98]	
*Within-group score change*	0.04 [−0.44, 0.52]	−0.03 [−0.48, 0.42]	0.34 [−0.20, 0.88]

Data are shown as mean [95% confidence interval for the mean] at baseline and post-intervention, and mean differences [95% confidence interval for the difference] for within- and between-group effects. Abbreviations: BPI: brief pain inventory; CI: confidence interval; PCS: pain catastrophizing scale; Significant between-group effect * *p* < 0.05; ^a^ Moderate effect size: Cohen d 0.6–0.8.

**Table 5:** Within- and between-group effects for subjective well-being, functioning and quality of life at baseline and post-intervention.

	HAMMAM Group (*n* = 21)	Control Group (*n* = 23)	Between-Group Effects
	Mean [CI(95%)]	Mean [CI(95%)]	
**Subjective well-being—EBS-20**			
**Satisfaction with life**			
*Baseline*	3.30 [2.78, 3.83]	3.25 [2.82, 3.69]	
*Post-intervention*	3.37 [2.87, 3.88]	3.38 [2.90, 3.86]	
*Within-group score change*	0.07 [−0.23, 0.37]	0.13 [−0.15, 0.40]	−0.06 [−0.45, 0.34]
**Positive affect**			
*Baseline*	3.79 [3.47, 4.11]	3.70 [3.36, 4.04]	
*Post-intervention*	3.63 [3.29, 3.97]	3.70 [3.33, 4.07]	
*Within-group score change*	−0.16 [−0.44, 0.12]	0.00 [−0.18, 0.17]	−0.16 [−0.47, 0.16]
**Total**			
*Baseline*	3.55 [3.16, 3.94]	3.48 [3.12, 3.83]	
*Post-intervention*	3.50 [3.11, 3.89]	3.54 [3.15, 3.93]	
*Within-group score change*	−0.05 [−0.20, 0.11]	0.06 [−0.13, 0.26]	−0.11 [−0.35, 0.14]
**Functioning—PROMIS-29**			
**Physical function**			
*Baseline*	16.00 [14.05, 17.95]	16.09 [14.64, 17.54]	
*Post-intervention*	15.90 [13.92, 17.89]	16.13 [14.74, 17.52]	
*Within-group score change*	−0.10 [−0.67, 0.48]	0.04 [−0.42, 0.50]	−0.14 [−0.85, 0.57]
**Pain intensity**			
*Baseline*	5.86 [4.88, 6.84]	5.96 [5.24, 6.68]	
*Post-intervention*	4.95 [3.69, 6.21]	6.09 [5.23, 6.94]	
*Within-group score change*	−0.90 [−2.03, 0.22]	0.13 [−0.60, 0.86]	−1.04 [−2.31, 0.24]
**Pain interference**			
*Baseline*	11.90 [10.29, 13.52]	11.96 [10.34, 13.58]	
*Post-intervention*	10.00 [7.75, 12.25]	12.30 [10.39, 14.22]	
*Within-group score change*	−1.90 [−3.51, −0.30]	0.35 [−0.74, 1.44]	−2.25 [−4.11, −0.40] *^a^
**Fatigue**			
*Baseline*	14.71 [13.10, 16.32]	15.00 [13.25, 16.75]	
*Post-intervention*	13.52 [11.79, 15.26]	14.22 [12.43, 16.01]	
*Within-group score change*	−1.19 [−2.75, 0.37]	−0.78 [−1.94, 0.38]	−0.41 [−2.27, 1.46]
**Sleep disturbance**			
*Baseline*	13.57 [11.93, 15.21]	12.22 [10.81, 13.62]	
*Post-intervention*	12.00 [10.62, 13.38]	12.30 [10.61, 14.00]	
*Within-group score change*	−1.57 [−3.39, 0.25]	0.09 [−1.14, 1.31]	−1.66 [−3.75, 0.43]
**Anxiety**			
*Baseline*	11.95 [10.65, 13.25]	12.65 [11.17, 14.13]	
*Post-intervention*	11.43 [9.58, 13.28]	12.48 [10.90, 14.05]	
*Within-group score change*	−0.52 [−1.80, 0.75]	−0.17 [−0.96, 0.62]	−0.35 [−1.78, 1.08]
**Depression**			
*Baseline*	10.81 [9.04, 12.58]	11.78 [10.30, 13.26]	
*Post-intervention*	9.52 [7.81, 12.24]	11.48 [9.64, 13.31]	
*Within-group score change*	−1.29 [−2.81, 0.24]	−0.30 [−1.55, 0.94]	−0.98 [−2.88, 0.92]
**Ability to participate in social roles and activities**			
*Baseline*	11.24 [9.61, 12.87]	11.65 [10.14, 13.16]	
*Post-intervention*	12.95 [11.19, 14.71]	12.09 [10.51, 13.66]	
*Within-group score change*	1.71 [0.40, 2.64]	0.43 [−0.45, 1.32]	1.09 [−0.28, 2.46]
**Quality of life—EHP-30**			
**Pain**			
*Baseline*	52.81 [43.83, 61.80]	57.61 [50.97, 64.25]	
*Post-intervention*	43.40 [32.88, 53.91]	50.40 [44.15, 56.64]	
*Within-group score change*	−9.42 [−16.44, −2.40]	−7.21 [−11.99, −2.44]	−2.20 [−10.31, 5.90]
**Control and powerlessness**			
*Baseline*	59.72 [49.66, 69.78]	69.57 [61.70, 77.43]	
*Post-intervention*	45.63 [34.39, 56.88]	55.80 [48.43, 63.16]	
*Within-group score change*	−14.07 [−22.84, −5.29]	−13.77 [−20.36, −7.17]	−0.30 [−10.83, 10.23]
**Emotional well-being**			
*Baseline*	55.75 [48.49, 63.01]	60.69 [53.80, 67.58]	
*Post-intervention*	50.40 [42.29, 58.51]	52.72 [47.06, 58.38]	
*Within-group score change*	−5.35 [−10.95, 0.25]	−7.97 [−13.87, −2.07]	2.62 [−5.32, 10.55]
**Social support**			
*Baseline*	59.23 [50.34, 68.12]	65.85 [57.68, 74.01]	
*Post-intervention*	53.87 [43.24, 64.49]	64.41 [57.53, 71.30]	
*Within-group score change*	−5.36 [−15.51, 4.79]	−1.43 [−10.46, 7.59]	−3.92 [−17.06, 9.21]
**Self-image**			
*Baseline*	59.13 [50.24, 68.02]	67.75 [58.14, 77.37]	
*Post-intervention*	52.78 [40.94, 64.61]	62.33 [53.64, 71.01]	
*Within-group score change*	−6.35 [−12.57, −0.13]	−5.43 [−12.60, 1.74]	−0.92 [−10.22, 8.38]
**Total**			
*Baseline*	56.27 [49.52, 63.02]	62.73 [57.28, 68.18]	
*Post-intervention*	47.58 [39.09, 56.07]	55.00 [50.09, 59.92]	
*Within-group score change*	−8.69 [−14.47, −2.91]	−7.73 [−12.17, −3.29]	−0.96 [−7.96, 6.03]

Data are shown as mean [95% confidence interval for the mean] at baseline and post-intervention, and mean differences [95% confidence interval for the difference] for within- and between-group effects. Abbreviations: CI: confidence interval; PROMIS-29: Patient-Reported Outcomes Measurement Information System-29; EBS-20: subjective well-being scale; EHP-30: endometriosis health profile; Significant between-group effect * *p* < 0.05; ^a^ Moderate effect size: Cohen d 0.6–0.8.

## Data Availability

The data that support the findings of this study are available from the corresponding author (F.A.-C.), upon reasonable request.

## Supplementary Materials

The following supporting information can be downloaded at: https://www.mdpi.com/article/10.3390/medicina60101677/s1, Table S1: Within- and between-group effects for pain interference at baseline and post-intervention.; Table S2: Within- and between-group effects for pressure pain thresholds at baseline and post-intervention.
