# Supplementary material for: The Benefits of an Integral HAMMAM Experience Combining Hydrotherapy and Swedish Massage on Pain, Subjective Well-Being and Quality of Life in Women with Endometriosis-Related Chronic Pelvic Pain: A Randomized Controlled Trial
Source: Medicina (Kaunas). 2024 Oct 13;60(10):1677. doi: 10.3390/medicina60101677 (PMC11509651; doi:10.3390/medicina60101677)
Supplement: Supplementary file 1 [file medicina-60-01677-s001.zip › medicina-3215454-supplementary.pdf]

Supplementary Table S1. Within- and between-group effects for pain interference at baseline and post-intervention.

	HAMMAM group (n=21)	Control group (n=23)	Between-Group Effects
	Mean [CI(95%)]	Mean [CI(95%)]	
<u>**Pain interference - BPI**</u>			
**Total**			
Baseline	5.08 [3.90, 6.25]	5.13 [4.16, 6.09]	
Post-intervention	3.94 [2.37, 5.51]	4.80 [3.46, 6.13]	
Within-group score change	-1.13 [-2.08, -0.18]	-0.33 [-1.58, 0.93]	-0.44 [-1.68, 0.80]
**General activity**			
Baseline	4.93 [3.84, 6.03]	5.82 [4.45, 7.20]	
Post-intervention	3.73 [2.09, 5.37]	5.24 [3.80, 6.67]	
Within-group score change	-1.20 [-2.42, 0.02]	-0.59 [-2.01, 0.83]	-0.68 [-2.16, 0.81]
**Mood**			
Baseline	5.87 [4.61, 7.12]	6.53 [5.52, 7.54]	
Post-intervention	4.53 [2.86, 6.21]	5.65 [4.26, 7.03]	
Within-group score change	-1.33 [-2.53, -0.14]	-0.88 [-2.07, 0.31]	-0.23 [-1.68, 1.23]
**Walking ability**			
Baseline	4.33 [2.92, 5.75]	4.41 [2.88, 5.94]	
Post-intervention	3.27 [1.55, 4.99]	4.24 [2.67, 5.80]	
Within-group score change	-1.07 [-2.55, 0.42]	-0.18 [-1.82, 1.47]	-0.16 [-1.83, 1.51]
**Normal work**			
Baseline	4.67 [3.24, 6.10]	4.82 [3.57, 6.07]	
Post-intervention	3.80 [2.00, 5.60]	4.65 [3.18, 6.11]	
Within-group score change	-0.87 [-1.97, 0.24]	-0.18 [-1.69, 1.34]	-0.15 [-1.70, 1.41]
**Relationships with other people**			
Baseline	4.93 [3.42, 6.45]	4.35 [3.12, 5.59]	
Post-intervention	3.73 [2.09, 5.37]	4.53 [3.03, 6.03]	
Within-group score change	-1.20 [-2.16, -0.24]	0.18 [-1.42, 1.77]	-0.80 [-2.29, 0.69]
**Sleep**			
Baseline	5.67 [4.06, 7.27]	4.24 [3.00, 5.48]	
Post-intervention	4.13 [2.40, 5.87]	4.59 [3.20, 5.97]	
Within-group score change	-1.53 [-2.79, -0.28]	0.35 [-1.11, 1.82]	-1.84 [-2.29, -0.33]*[a]
**Enjoyment of life**			
Baseline	5.13 [3.55, 6.71]	5.71 [4.67, 6.75]	
Post-intervention	4.40 [2.42, 6.38]	4.71 [2.94, 6.48]	
Within-group score change	-0.73 [-1.81, 0.34]	-1.00 [-2.63, 0.63]	0.77 [-0.91, 2.45]

Data are shown as mean [95% confidence interval for the mean] at baseline and post-intervention, and mean differences [95% confidence interval for the difference] for within- and between-group effects. Abbreviations: BPI: brief pain inventory; CI: confidence intervals; Significant between-group effect *P<0.05; [a]Moderate effect size: Cohen d 0.6-0.8.

Supplementary Table S2. Within- and between-group effects for pressure pain thresholds at baseline and post-intervention.

	**HAMMAM group (n=21)**	**Control group (n=23)**	
	**CI(95%)**	**CI(95%)**	**Between-Group Effects**
<u>**Pressure pain thresholds - Algometry**</u>			
**Supraumbilical, right side**			
Baseline	1.57 [1.17, 1.98]	1.55 [1.09, 2.02]	
Post-intervention	1.61 [1.11, 2.10]	1.29 [0.90, 1.68]	
Within-group score change	0.03 [-0.39, 0.46]	-0.26 [-0.80, 0.28]	0.23 [-0.29, 0.75]
**Infraumbilical, right side**			
Baseline	1.26 [0.85, 1.68]	1.17 [0.79, 1.55]	
Post-intervention	1.43 [0.96, 1.90]	1.06 [0.72, 1.41]	
Within-group score change	0.16 [-0.21, 0.53]	-0.10 [-0.41, 0.20]	0.34 [-0.05, 0.72]
**Supraumbilical, left side**			
Baseline	1.49 [1.06, 1.91]	1.50 [1.06, 1.95]	
Post-intervention	1.58 [1.03, 2.13]	1.19 [0.76, 1.63]	
Within-group score change	0.09 [-0.46, 0.65]	-0.31 [-0.88, 0.27]	0.36 [-0.26, 0.97]
**Infraumbilical, left side**			
Baseline	1.33 [0.90, 1.75]	1.04 [0.71, 1.37]	
Post-intervention	1.43 [0.91, 1.95]	1.06 [0.69, 1.43]	
Within-group score change	0.10 [-0.34, 0.54]	0.03 [-0.24, 0.29]	0.16 [-0.23, 0.56]
**Pubis symphysis**			
Baseline	1.17 [0.75, 1.59]	1.10 [0.68, 1.52]	
Post-intervention	1.54 [1.08, 2.00]	1.00 [0.62, 1.38]	
Within-group score change	0.37 [-0.09, 0.83]	-0.10 [-0.46, 0.26]	0.40 [-0.05, 0.85]
**Inguinal ligament, right side**			
Baseline	1.23 [0.74, 1.72]	1.06 [0.62, 1.51]	
Post-intervention	1.46 [1.00, 1.91]	1.01 [0.60, 1.42]	
Within-group score change	0.23 [-0.21, 0.66]	-0.05 [-0.37, 0.26]	0.32 [-0.06, 0.70]
**Inguinal ligament, left side**			
Baseline	0.90 [0.62, 1.19]	1.00 [0.61, 1.38]	
Post-intervention	1.48 [1.05, 1.91]	0.99 [0.59, 1.38]	
Within-group score change	0.58 [0.22, 0.94]	-0.01 [-0.36, 0.34]	0.57 [0.18, 0.96][*b]
**Lumbar, right side**			
Baseline	2.00 [1.25, 2.74]	2.23 [1.48, 2.98]	
Post-intervention	2.31 [1.61, 3.00]	2.53 [1.55, 3.51]	
Within-group score change	0.31 [-0.21, 0.84]	0.30 [-0.36, 0.95]	0.18 [-0.56, 0.92]
**Lumbar, left side**			
Baseline	1.84 [1.09, 2.60]	2.31 [1.46, 3.15]	
Post-intervention	2.49 [1.69, 3.29]	2.59 [1.34, 3.84]	
Within-group score change	0.65 [-0.14, 1.43]	0.28 [-0.52, 1.09]	0.41 [-0.47, 1.29]
**Second metacarpal, right side**			
Baseline	2.16 [1.49, 2.82]	2.16 [1.58, 2.73]	
Post-intervention	2.21 [1.50, 2.92]	2.25 [1.37, 3.13]	

Within-group score change	0.05 [-0.42, 0.53]	0.09 [-0.41, 0.60]	0.19 [-0.42, 0.79]
**Second metacarpal, left side**			
Baseline	2.17 [1.41, 2.92]	2.21 [1.58, 2.84]	
Post-intervention	2.19 [1.40, 2.99]	2.06 [1.26, 2.87]	
Within-group score change	0.03 [-0.51, 0.56]	-0.15 [-0.61, 0.31]	0.48 [-0.08, 1.04]

Data are shown as mean [95% confidence interval for the mean] at baseline and post-intervention, and mean differences [95% confidence interval for the difference] for within- and between-group effects. Abbreviations: CI: confidence intervals; Significant between-group effect *P<0.05; [b]Large effect size: Cohen d>0.8.
